# The cAMP Inducers Modify *N*-Acetylaspartate Metabolism in Wistar Rat Brain

**DOI:** 10.3390/antiox10091404

**Published:** 2021-09-01

**Authors:** Robert Kowalski, Piotr Pikul, Krzysztof Lewandowski, Monika Sakowicz-Burkiewicz, Tadeusz Pawełczyk, Marlena Zyśk

**Affiliations:** 1 University Clinical Center in Gdansk, 80-952 Gdansk, Poland; robert.kowalski@gumed.edu.pl (R.K.); krzysztof.lewandowski@gumed.edu.pl (K.L.); 2 Laboratory of Molecular and Cellular Nephrology, Mossakowski Medical Research Institute, Polish Academy of Sciences, 80-308 Gdansk, Poland; piotr.pikul@gumed.edu.pl; 3 Department of Laboratory Medicine, Medical University of Gdansk, 80-210 Gdansk, Poland; 4 Department of Molecular Medicine, Medical University of Gdansk, 80-210 Gdansk, Poland; monika.sakowicz-burkiewicz@gumed.edu.pl (M.S.-B.); tadeusz.pawelczyk@gumed.edu.pl (T.P.)

**Keywords:** NAT8L, primary neurons, neural stem cells

## Abstract

Neuronal *N*-acetylaspartate production appears in the presence of aspartate *N*-acetyltransferase (NAT8L) and binds acetyl groups from acetyl-CoA with aspartic acid. Further *N*-acetylaspartate pathways are still being elucidated, although they seem to involve neuron-glia crosstalk. Together with *N*-acetylaspartate, NAT8L takes part in oligoglia and astroglia cell maturation, myelin production, and dopamine-dependent brain signaling. Therefore, understanding *N*-acetylaspartate metabolism is an emergent task in neurobiology. This project used in in vitro and in vivo approaches in order to establish the impact of maturation factors and glial cells on *N*-acetylaspartate metabolism. Embryonic rat neural stem cells and primary neurons were maturated with either nerve growth factor, *trans*-retinoic acid or activators of cAMP-dependent protein kinase A (dibutyryl-cAMP, forskolin, theophylline). For in vivo, adult male Wistar rats were injected with theophylline (20 mg/kg b.w.) daily for two or eight weeks. Our studies showed that the *N*-acetylaspartate metabolism differs between primary neurons and neural stem cell cultures. The presence of glia cells protected *N*-acetylaspartate metabolism from dramatic changes within the maturation processes, which was impossible in the case of pure primary neuron cultures. In the case of differentiation processes, our data points to dibutyryl-cAMP as the most prominent regulator of *N*-acetylaspartate metabolism.

## 1. Introduction

Aspartate *N*-acetyltransferase (NAT8L) is a neuronal enzyme producing *N*-acetylaspartate (NAA) from aspartate and acetyl-CoA [1,2]. In brain cells, acetyl-CoA is produced either from pyruvate (in the presence of pyruvate dehydrogenase) or is a final product of fatty acid β-oxidation [3]. In mitochondria, acetyl-CoA fuels the tricarboxylic acid cycle, which together with the electron transport chain, constitutes the main energy source [3]. Aspartate, the second substrate for NAA production, drifts along the aspartate—malate shuttle [1,2]. Thus, in brain MRI scans, NAA is a marker of neuronal metabolic condition, although the exact function of NAA remains unsure [2]. Thus, so far, it has been shown that NAA is a messenger conducting neuronal crosstalk with oligodendrocytes and astrocytes [1,4,5]. Once oligodendrocytes have taken up the NAA, they utilize it to produce myelin and energy [1,4,5]. Astrocytes meanwhile incorporate NAA derivates into glutamine metabolism [1,4,5].

MRI scans of human brains have revealed significant disruptions of NAA levels in several brain diseases [6]. The reduction of *N*-acetylaspartate concentration has been reported in Alzheimer’s disease, Parkinson’s disease, multiple sclerosis, autism, and adult depression [1,2,4,7,8,9,10,11,12]. Recent studies suggest that the mechanism of this pathology is probably linked with the biological activity of aspartate *N*-acetyltransferase (NAT8L). It has been shown that aspartate *N*-acetyltransferase level fluctuates during mammalian life. In particular, *Nat8l* mRNA level is low in the murine embryonal brain, but once the fetus is born the *Nat8l* mRNA level dramatically increases [4]. Furthermore, even adult mice do not reach a plateau in *Nat8l* mRNA level [4]. Studies with methamphetamine have considered cAMP-dependent protein kinase A (PKA) as an enzyme upregulating the *N*-acetylaspartate level [13,14,15]. However, the published studies were conducted mainly using immature neuroblastoma cells or juvenile animals entering adulthood [13,14,15]. Our previous studies investigating the impact of cholinergic neurotransmission showed that maturation of cholinergic SN56 neuroblastoma cells by db-cAMP and *trans*-retinoic acid decreased NAA level and increased aspartate *N*-acetyltransferase level [16,17]. The same maturation approach introduced to SH-SY5Y human neuroblastoma cells also decreased NAA level [17]. Since db-cAMP treatment has been showed to increase protein kinase A activity followed by CREB-dependent neuronal maturation, our data suggested CREB-dependent depletion of the NAA level, which is an opposite mechanism than that observed in methamphetamine studies [13,15,16,17]. Thus, in this study, using different research models, we considered the impact of maturation factors on NAA metabolism. Our working hypothesis was that the brain cells maturation processes might regulate *N*-acetylaspartate metabolism. For the purposes of this study, we used embryonic rat neural stem cells (maturated with either nerve growth factor or *trans*-retinoic acid or dibutyryl-cAMP, forskolin or theophylline), primary neurons (maturated with nerve growth factor or dibutyryl-cAMP) and adult male Wistar rats injected daily with theophylline, 20 mg/kg b.w. for two or eight weeks.

## 2. Materials and Methods

### 2.1. Materials

Unless otherwise specified, all used compounds were specified at Supplement 1, while cell culture disposables were provided by Sarstedt (Blizne Łaszczyńskiego, Poland). Unless otherwise specified, spectrophotometric assays were run either using Ultraspec 3100 Pro (Amersham Biosciences, Warsaw, Polnad) or, for multiple well plate-based assays, Victor3, 1420 Multilabel Counter (Perkin Elmer, Warsaw, Poland).

### 2.2. Animals (In Vivo Studies)

All the experiments have been approved by the Polish Bioethics Committee (44/2016, 23 November 2016, Bydgoszcz, Poland). Studies followed the EU Directive 2010/63/EU and the International Council for Laboratory Animal Science guidelines for animal experiments. 

White male adult Wistar rats were housed at the Animal House (Medical University of Gdansk) with access to food and water ad libitum under 12 h standard light/dark cycle. The rats’ average weight before the experiments was 180–230 g followed by 200–350 g of weight reached at the end of the experiments. For this study purpose, animals were divided randomly to different treatment groups with the following group size: sham control group (2 weeks)—11 male rats, 2 weeks theophylline—8 male rats, sham control group (8 weeks)—6 male rats, 8 weeks theophylline—6 male rats.

**Figure 1:**
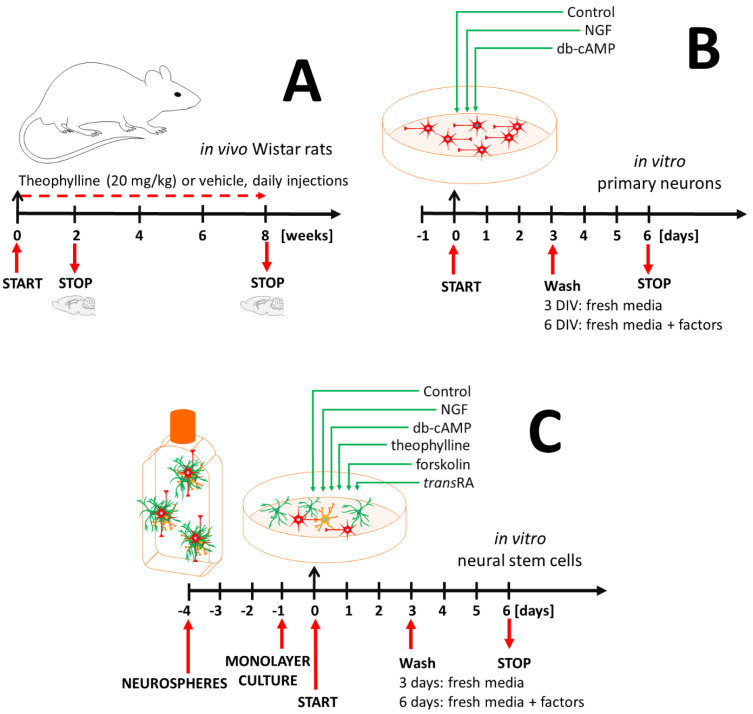
Experimental workflow: (**A**) Wistar rats challenged with theophylline (20 mg/kg b.w.) for 2 or 8 weeks; (**B**) primary neurons maturated with nerve growth factor or db-cAMP; (**C**) neural stem cells culture as neurospheres followed by monolayer culture and treated with maturation factors.

A total of 20 mg/kg of theophylline/0.9% NaCl was administrated on a daily basis (i.p.) for 2 or 8 weeks before sacrifice (Figure 1A). The sham control group was treated with a similar volume of NaCl without theophylline (Figure 1A). The animals were euthanized by the pentobarbitone overdose (2 mL/kg b.w., i.p., concentration: 0.66 M) [17]. 

### 2.3. Primary Cultures (In Vitro Studies)

Embryonal brain cortices (E18) obtained from pregnant Wistar rats (RRID: RGD_13508588) were dissected in Hank’s buffered salt solution (HBSS) supplemented by 8 mM HEPES (pH = 7.4), 100 U/mL penicillin and streptomycin. The single cell suspension was used further for either primary neuronal culture (PR) or neural stem cells culture (NSC) [18].

### 2.4. Embryonic Primary Neurons (PR)

Isolated cells were seeded as a monolayer in poly-L-ornithine and laminin coated dishes in a neurobasal medium supplemented with B27 supplement, 2 mM Glutamax as well as 100 U/mL penicillin and streptomycin. At the following day, the culture media were exchanged to control culture media (neurobasal medium supplemented with B27 supplement, 2 mM Glutamax, 20 nM AraC, 100 U/mL penicillin plus streptomycin) with or without maturating factors described below (Figure 1B). The contamination of primary neurons was controlled β-III-tubulin (neurons), GFAP (astrocytes), and CNPase (oligodendrocytes) specific markers (Table 1) [18].

### 2.5. Embryonic Neural Stem Cells (NSC)

Dissected cortices were cultured as neurospheres in DMEM/F12-GlutaMAX supplemented with B27 supplement, 10 ng/mL basic fibroblast growth factor, 20 ng/mL epidermal growth factor and 100 U/mL penicillin plus streptomycin with passaging every third day of culture. Cells between 1–3 passages were considered as suitable for these studies. For the experimental stage, cells were seeded as a monolayer in poly-L-ornithine and laminin coated dishes and left for 24 h in the same media as were used for the neurosphere culture. Next, the neural stem cells (NSC) were cultured in culture media (DMEM/F12-GlutaMAX supplemented with B27 supplement and 100 U/mL penicillin plus streptomycin) with or without the maturating factors described below (Figure 1C). Subpopulations of different cell types in (NSC) were recognized by β-III-tubulin (neurons), GFAP (astrocytes), and CNPase (oligodendrocytes) specific markers (Table 1) [18].

### 2.6. Treatment Strategy

The experimental setup was settled for 3 or 6 days in vitro (DIV) (Figure 1B,C). Primary cells were treated by different differentiation factors:

(A) control (no maturating factor);
(B) 10 ng/mL nerve growth factor (nerve growth factor—dependent differentiation pathway) [19];
(C) 1 mM dibutyryl-cAMP (cAMP—protein kinase A—dependent CREB pathway activation) [20];
(D) 10 µM theophylline (cAMP—protein kinase A—dependent CREB pathway activation) [20];
(E) 20 µM forskolin (cAMP—protein kinase A—dependent CREB pathway activation) [20].
(F) 1 µM *trans*-retinoic acid (retinoic acid-dependent CRAB pathway activation) [16].

Concentrations of maturating factors were chosen according to the literature reporting them as suitable for our cell culture approach.

### 2.7. Sample Preparation

Brain tissues (without cerebellum) or cells were homogenized in chilled: 4% HClO_4_ (for metabolic studies); 0.1 M HCl (for NAD assay); 0.2 M KOH (for NADH assay); 5% metaphosphoric acid (for glutathione assays); or buffer 5 mM HEPES (pH = 7.4) with 0.32 M sucrose and 0.1 mM EDTA (for enzymatic assays). After centrifugation at 13,000× *g* (4 °C, 15 min), each sample was immediately used for studies or kept at −80 °C until analyzed.

### 2.8. Enzymatic Assays

To analyze enzymatic activity in the cell lines, from each dish two independent cell lysates were collected and reported as a one average result. To analyze enzymatic activity in brain tissue, each of 3 tissue lysates was measure in two independent wells. A total of 6 achieved results were reported as 1 average activity. To analyze enzymatic activity in cell homogenate, each lysate was measure in two independent wells. Further, 2 achieved results were reported as 1 average activity. Protocols with details for enzymatic assays are shown in Supplement 2. 

Aconitase (EC 4.2.1.3), aspartate aminotransferase (GOT, EC 2.6.1.1), aspartate *N*-acetyltransferase (NAT8L, EC 2.3.1.17), choline acetyltransferase (ChAT, EC 2.3.1.6), citrate synthase (SC, EC 2.3.3.1), isocitrate dehydrogenase (IDH, EC 1.1.1.42), lactate dehydrogenase/LDH in media assay (LDH, EC 1.1.1.27), and pyruvate dehydrogenase (PDHC, EC 1.2.4.1) were used. Detailed descriptions of these methods can be found elsewhere [17].

### 2.9. Metabolic Assays

The results were calculated from the same number of samples for enzymatic assays. Protocols with details for metabolic profile assays are shown in Supplement 2.

Acetyl-CoA, aspartate, ATP, ADP, adenosine [21], β-hydroxybutyrate, reduced and oxidized glutathione [22], lactate, *N*-acetylaspartate, NAD and NADH [23], oxaloacetate, pyruvate, TBARS. Detailed descriptions of these methods can be found elsewhere [17].

### 2.10. Morphology Imaging and Analysis by NeuronJ (ImageJ Plugin)

Images were captured under 40× magnification using inverted light microscope (Aviovert 25, Zeiss) [16]. The cell morphology and cell-cell contact were measured with NeuronJ ImageJ macro and quantified using Pemberton and colleagues’ protocol [24]. In total, data were analyzed from 3 independent experiments. Image pre-processing assessment included: image sharpening, contrast enhancing, binary image production and image skeletonization. The NeuronJ image processing reports branch length and cell appendages normalized to the controls.

### 2.11. Proliferation and Viability Assays

Protocols with details for proliferation assays are detailed in Supplement 2.

Further, the 6-CFDA test [25], acid phosphatase test [26], DAF-2 DA intracellular nitric oxide test [27], LDH in media test [17], MTT test [28], xCELLigence real time cell analysis were used [29].

### 2.12. Real-Time RT-qPCR Analysis of NAT8L and Chat mRNA Levels

The 2 × 10^6^ cells or 0.1 g brain tissues were vortexed or homogenized in a sterile tube with 0.5 mL (cells) or 1 mL (brain tissue) of RNA Extracol extraction buffer (Eurx, Cat #E3700-02). The extraction was incited by the addition of 250 μL chloroform (per 1 mL of RNA Extracol buffer). After vigorous shaking, each sample was incubated at 4 °C for 15 min and spun down (10,000× *g* for 15 min at 4 °C). The upper aqueous phase was transferred to a new tube and refilled by isopropanol (POCH, Cat #751500111) in ratio 1:2 (isopropanol: RNA Extracol, *v*/*v*). The RNA precipitation was carried out overnight at −20 °C and on the following day each sample was centrifuged (10,000× *g* for 15 min at 4 °C). RNA pellet was washed firstly with 99.8% and then with 75% (*v*/*v*) ethanol, air-dried and reconstituted in nuclease-free water (15–20 μL) (Sigma Aldrich, Cat# W4502). The obtained samples were kept at −20 °C until needed. The quantity of isolated RNA was determined by fluorometry, using the Qubit RNA HA assay kit according to the manufacturer’s instructions (ThermoFisher Sc., Cat #Q32855). The gene expression levels of *Nat8l* encoding NAT8L enzyme and *Chat* encoding ChAT enzyme were determined by real-time RT-qPCR performed in a Light Cycler 480 II (Roche Diagnostic GmbH, Germany) using Path-IDTM Multiplex One-Step RT-PCR Kit (ThermoFisher Sc., Cat #4442135) and Universal ProbeLibrary for Rat, and gene-specific intron-spanning primers (Table 2). The reaction mixture in the final volume 10 μL contained 5 μL of Multiplex RT-PCR Buffer, 1 μL of Multiplex Enzyme Mix and 0.5 μL of each primer for target transcript, 0.2 μL of a target probe, 0.2 μL of primers’ reference gene, 0.2 μL of probe for reference transcript and 2 μL of total RNA (Table 2). The target gene transcript levels were normalized to reference transcript of the β-actin gene (Actb). Reverse transcription program: 48 °C—10 min, and 95 °C—10 min. The amplification program was as follows: 95 °C—15 s, 60 °C—45 s for 45 cycles. Data were processed with Light Cycler 480 II software 2.0 [17].

### 2.13. Western Blot Analysis

The cells or brain tissues (without cerebellum) were lysed for 30 min in lysis buffer (1% protease inhibitor cocktail, 50 mM Tris-HCl buffer pH 7.4, 5 mM EDTA, 100 mM NaCl, 1% Triton-X100, 5% glycerol, 10 mM KH_2_PO_4_), at 4 °C. The obtained lysates were kept at −20 °C until analysis. Each sample (40 µg of protein)/20 µL of 10 mM dithiothreitol/Laemmli buffer) was incubated for 1 h at 57 °C followed by the addition of 2 µL 500 mM 2-chloroacetamide and incubation for 1 h at room temperature. Next, the spun down samples were loaded to ExpressPlusTM PAGE Gel 4—20% gradient BisTris-PAGE gels (GenSignal, Cat #GS1960). Then, the BisTris-PAGE gel was run at 300 V for 17 min in MOPS/SDS running buffer (1 M Tris pH = 7.7, 1 M MOPS, 70 mM SDS, 20.5 mM EDTA) in a MINI-PROTEAN electrophoresis system with cooling (Roche, Germany). Next, proteins were transfer from Bis Tris-PAGE gel to a PVDF membrane (pore size: 0.2 µm, iBlot^®^ transfer stack, Cat #IB401001) using iBlot^®^ Dry Blotting System with P0 program (program details: 1 min—20 V, 4 min—23 V, 3 min—25 V) (ThermoFisher, Munich, Germany). The PVDF membrane was washed 2 × 10 min in TBTS buffer (25 mM Tris-HCl pH = 7.4, 135 mM NaCl, 3 mM KCl, 0.5% Tween20). Non-specific bindings were blocked with 5% BSA in TBST (60 min, room temperature). Next, the PVDF membrane was incubated with specific primary antibodies (1:500, *v*/*v*) in 5% BSA/TBST buffer (4 °C, overnight) (Table 1). On the following day, after 4 × 10 min washing, the membrane was incubated with polyclonal AP—conjugated secondary antibodies diluted 1: 5000 (*v*/*v*) with 5% BSA/TBST buffer (3 h, room temperature) (Table 1). PVDF membrane was developed in a dark room for 15 min with developing buffer pH = 9.5 (0.1 M Tris buffer, 0.1 M NaCl, 5 mM MgCl_2_, 0.33 mg/mL nitrotetrazolium blue chloride and 0.17 mg/mL BCIP) [17,30].

### 2.14. Protein Assay

Protein was assayed by the method of Bradford [31] with human immunoglobulin as a standard.

### 2.15. Statistics

The Kolmogorov–Smirnov normality test exclude the normal data distribution. Therefore, the results were tested by either the Mann–Whitney test or Kruskal–Wallis followed by Dunn’s multiple comparison post-test, where values of *p* < 0.05 were considered statistically significant. The data are presented as a median with ranges (25th–75th percentile). All statistical analyses were performed using the Graph Pad Prism 4.0 statistical package (Graph Pad Software, San Diego, CA, USA).

## 3. Results

### 3.1. Differentiation Factors Did Not Affect Neural Stem Cell Viability

When considering the main maturation pathways of brain cells, we chose the following factors: nerve growth factor activating a tropomyosin receptor kinase-dependent PKB/PKC pathway (differentiation factor targeting neurons) [32]; *trans*-retinoic acid activating a CRABP-dependent differentiation pathway activator [33]; and dibutyryl-cAMP/theophylline/forskolin (activators of a cAMP/PKA-dependent CREB pathway) [34]. Nerve growth factor (NGF) is known to be neuron-targeting protein driving genes responsible for neuron maturation and survival [32]. In contrast, dibutyryl-cAMP (db-cAMP), forskolin, and theophylline activate the protein kinase A followed by the activation of CREB dependent gene expression [20]. The protein kinase A-dependent pathway occurs in any kind of cells, so unlike the NGF treatment, cAMP differentiates all neural stem cell sub-populations. Neural stem cells culture includes neural-origin cells, in particular: astrocytes (70–80% of the cell population), neurons (20–25%) and less that 10% of oligodendrocytes (Supplement 3 Figure S1A–C) and do not contain microglia (known to have hematopoiesis origin) [18]. The contamination of primary neurons with glia cells reached 5–10% in each cell culture (Supplement 3 Figure S1D,E). 

**Figure 2:**
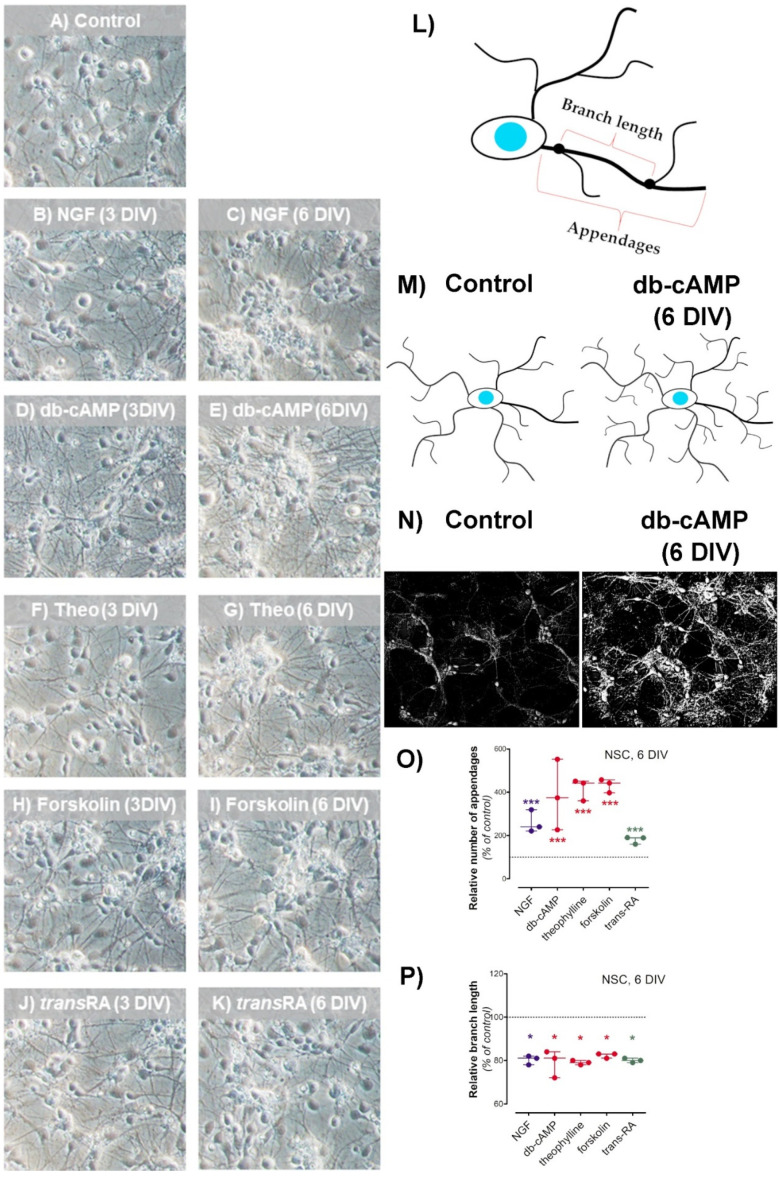
Impact of differentiation factors on neural stem cell (NSC) morphology was analyzed with the following factors: (**A**) Control; (**B**,**C**) nerve growth factor (NGF, 10 ng/mL); (**D**,**E**) dibutyryl-cyclic AMP (db-cAMP, 1 mM); (**F**,**G**) theophylline (Theo, 10 µM); (**H**,**I**) forskolin (20 µM); (**J**,**K**) *trans*-retinoic acid (transRA, 1 µM); Images are representative for 4 independent experiments. The cell morphology analysis was conducted after 6 DIV with Neuron J plugin (from ImageJ): (**L**)—cartoon definition of cell appendages and branches; (**M**)—cartoon visualization of cell morphology cultured without and with growth factors; (**N**)—ImageJ processed images of NSC cultured without and with growth factors; (**O**)—the number of cell appendages significantly improves after 6 DIV culture with growth factors; (**P**)—the average branch length decreased in NSC culture treated with growth factors. Results are showed as medians with interquartile ranges from 3 observations per group. Significantly different from the control: * (*p*-value < 0.05), *** (*p*-value < 0.001). Abbreviations: 3 DIV: 3 days of culture in media with differentiation factors plus 3 days of factor-free culture; 6 DIV: 6 days of culture in media with differentiation factors.

**Figure 3:**
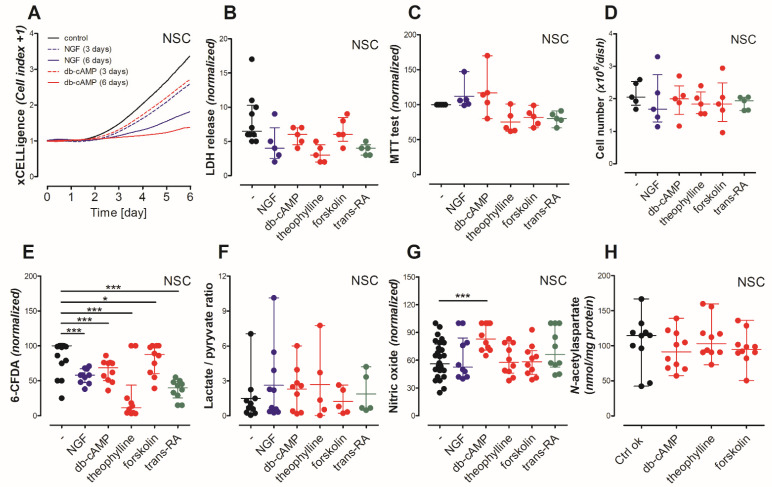
Impact of maturating factors on neural stem cell viability (6-day treatment): (**A**) xCELLigence real time measurement; (**B**) LDH in media test; (**C**) MTT test; (**D**) total number of cells; (**E**) 6-CFDA assay; (**F**) lactate/pyruvate ratio; (**G**) nitric oxide production; (**H**) *N*-acetylaspartate level. Results are shown as medians with interquartile ranges from 3–11 observations per group. (**B**,**C**,**E**,**G**): results were normalized according to the highest value in particular experiment. Results are showed as medians with interquartile ranges from 5–23 observations per group. Significantly different from the control: * (*p*-value < 0.05), *** (*p*-value < 0.001). Abbreviations: db-cAMP: dibutyryl-cyclic AMP, NGF: nerve growth factor, *trans*-RA: *trans*-retinoic acid.

During 6 days of cell culture, control neural stem cells (NSC) remained equally spread across the dish surface area. Meanwhile, the differentiated NSC grew into cell clusters (Figure 2A–K). The xCELLigence real time monitoring system showed that differentiated NSC, by growing into clusters, covered a considerably less dish surface area than untreated cells (Figure 3A). The first cell clusters were noted after 3 days of treatment by either dibutyryl-cAMP (db-cAMP) or forskolin (Figure 2D,E,H,I). NSC treated by either nerve growth factor (NGF), theophylline or *trans*-retinoic acid (*trans*RA) needed at least 3 days more to organize cell clusters (Figure 2B,C,F,G,J,K). 

To quantify the changes in cell morphology, we used NeuronJ plugin from ImageJ (Figure 2L–P). Image processing showed the NSC cell clusters connected with each other by a complex network characterized by increased number of cell appendages and decreased branch length (Figure 2N–P). A total cell number and 6-CFDA tracer did not indicate any differences in proliferation rate between the used maturation approaches (Figure 3D,E). Interestingly, the fluorescence signal coming from NSC cultured with theophylline or *trans*-retinoic acid followed by 6-CFDA staining was dramatically decreased, which was not supported by cell number counting (Figure 3D,E). Considering the cellular metabolism of these growth factors and 6-CFDA diffusion into the cell mechanism, it occurred that all compounds need estereses involvement into their cellular metabolism [35,36]. Thus the 6-CFDA assay limitation required additional viability studies to confirmed non-toxic impact of growth factors on NSC. No maturation factors affected NSC viability, as the lactate dehydrogenase in media and MTT tests showed (Figure 3B,C). Furthermore, astrocytes did not increase lactate/pyruvate ratio or the nitric oxide production, which might suggest that they stayed in a non-activated state (Figure 3F,G). Viability and proliferation assays did not show any particular differences between PKA activators (db-cAMP, forskolin, theophylline) (Figure 3B–G). Furthermore, the *N*-acetylaspartate level was not differ between these activators, therefore, in further study db-cAMP was used as a membrane permeant version of cAMP (Figure 3H).

### 3.2. Differentiation Factors Moderated Primary Cell Culture Energy Metabolism

**Figure 4:**
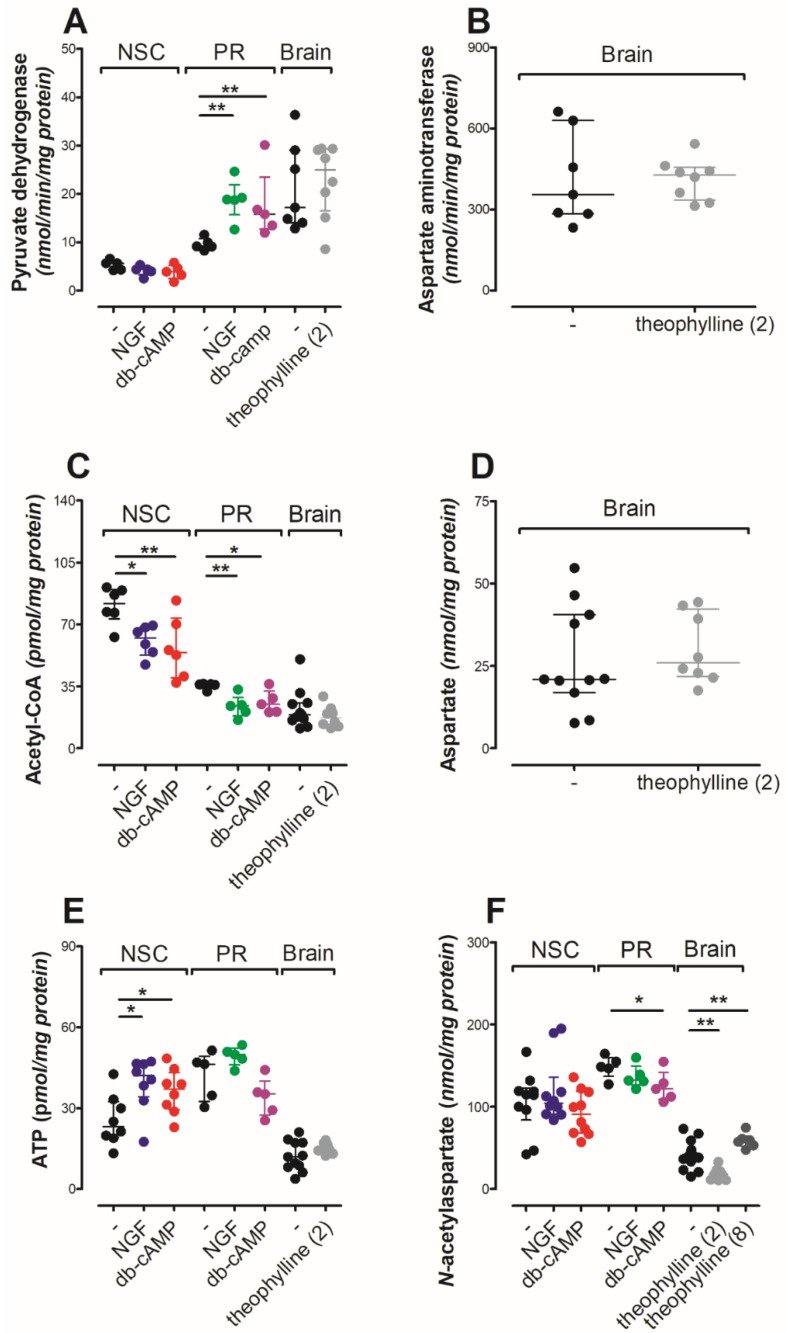
Impact of differentiation factors on NAA-related markers. (**A**) Pyruvate dehydrogenase activity; (**B**) aspartate aminotransferase activity; (**C**) acetyl-CoA level; (**D**) aspartate level; (**E**) ATP level; (**F**) *N*-acetylaspartate level. Results are showed as medians with interquartile ranges from 5–10 observations per group. Significantly different from the control: * (*p*-value < 0.05), ** (*p*-value < 0.01). Abbreviations: db-cAMP: dibutyryl-cyclic AMP, NGF: nerve growth factor, NSC: neural stem cells; PR: primary neurons; theophylline (2): 2 weeks theophylline treatment; theophylline (8): 8 weeks theophylline treatment.

Mature neurons prefer mitochondrial energy metabolism instead of glycolysis [37]. This metabolic shift (tracked by pyruvate dehydrogenase (PDH) activity, acetyl-CoA and pyruvate levels) was clearly observed in primary neurons treated by either NGF or db-cAMP (Table 3, Figure 4A,C). Since neurons are a minority in the neural stem cell culture, the metabolic changes remained undetectable (Table 3, Figure 4A). The NGF or db-cAMP treatment increased ATP (* *p* < 0.05) and decreased acetyl-CoA (* *p* < 0.05) levels in NSC cells (Figure 4C,E), which suggested significant metabolic changes in cell culture. The differentiation of both primary neurons and NSC did not affect lactate dehydrogenase (LDH) activity, although primary neuron maturation significantly increased the lactate/pyruvate ratio (Table 3 and Table 4) (* *p* < 0.05). The comparison of NSC with primary neurons revealed significant differences between these cell cultures. Comparing the NSC and primary neurons, both cell lines maintained comparable ATP, especially the growth factor treated cells (Figure 4E). However, the primary neurons present a significantly higher energy metabolism rate as shown by significantly higher LDH and PDH activities as well as significantly lower pyruvate, acetyl-CoA and lactate levels (Table 3 and Table 4, Figure 4A,C) (*** *p* < 0.001). The nerve factor treatment did not affect aconitase activity in any experimental points (Table 4). In contrast, db-cAMP treatment inhibited isocitrate dehydrogenase activity in NSC, but not in primary neurons (Table 4).

### 3.3. Acute Theophylline Treatment Did Not Affect Brain Energy State 

To study cAMP—dependent protein kinase A activation using the in vivo model, we considered daily acute theophylline challenge instead of db-cAMP treatment. Theophylline injection has been proven to have a spread effect across the entire animal body (including the brain), while db-cAMP might not reach brain tissue, unless microinjected into the particular brain region [37]. 

**Figure 5:**
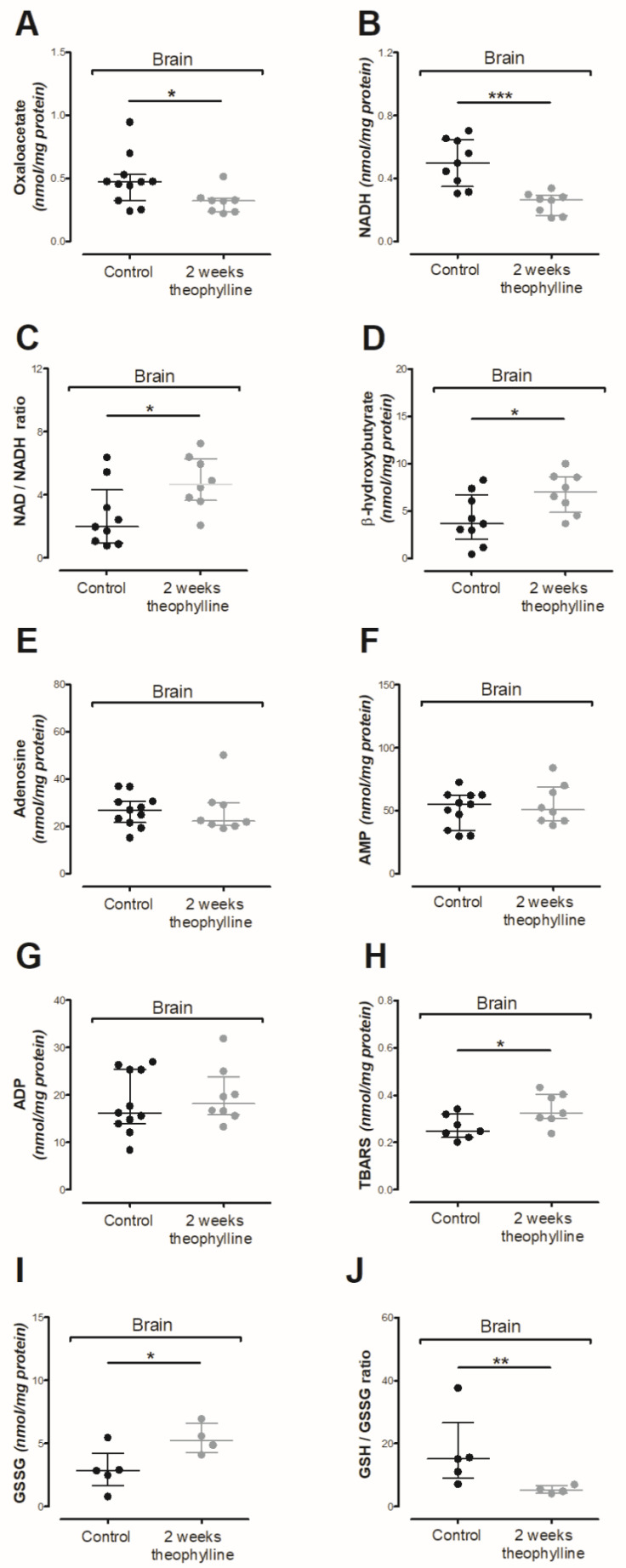
Impact of theophylline treatment on Wistar rat brain: (**A**) oxaloacetate level; (**B**) reduced nicotinamide adenine dinucleotide level (NADH) level; (**C**) NADH/NAD ratio; (**D**) β-hydroxybutyrate level; (**E**) adenosine level; (**F**) AMP level; (**G**) ADP level; (**H**) TBARS level; (**I**) oxidized glutathione (GSSG) level; (**J**) GSSG/GSH ratio. Data are medians with interquartile ranges from 3–11 observations per group. Significantly different from the control: * (*p*-value < 0.05), ** (*p*-value < 0.01), *** (*p*-value < 0.001).

**Figure 6:**
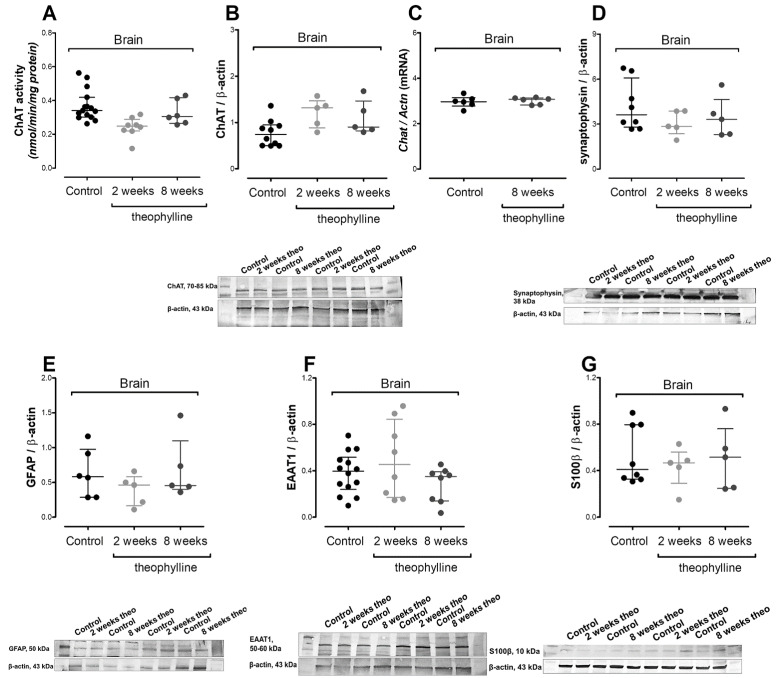
Impact of theophylline treatment on Wistar rat brain: (**A**) choline acetyltransferase (ChAT) activity; (**B**) choline acetyltransferase (ChAT) protein level; (**C**) choline acetyltransferase (*Chat*) mRNA level; (**D**) synaptophysin protein level; (**E**) glial fibrillary acidic protein (GFAP) level; (**F**) excitatory amino acid transporter 1 (EAAT1) protein level; (**G**) calcium binding protein β (S100β) protein level. Results are showed as medians with interquartile ranges from 5–15 observations per group.

A 2-week theophylline treatment did not affect pyruvate dehydrogenase (PDH) activity or its substrate (pyruvate), although lactate dehydrogenase and citrate dehydrogenase were significantly upregulated (Table 4, Figure 4A) (* *p* < 0.05). The overactivity of citrate dehydrogenase was achieved with a significantly lower oxaloacetate level, while upregulated lactate dehydrogenase activity did not increase lactate level (Table 3 and Table 4, Figure 5A) (* *p* < 0.05). Similar as with NSC, the intracellular accumulation of cAMP did not affect aconitase, although isocitrate dehydrogenase activity was significantly decreased (Table 4) (* *p* < 0.05). In contrast to aconitase, isocitrate dehydrogenase converted NAD into NADH, which after theophylline treatment was significantly decreased as the NAD/NADH ratio (Figure 5B,C) (* *p* < 0.05, *** *p* < 0.001). Consequently, disturbances in the NAD/NADH ratio increased the β-hydroxybutyrate level (Figure 5D) (* *p* < 0.05). Considering the inhibition effect of theophylline on phosphodiesterase activity, we analyzed the levels of ATP, ADP, AMP and adenosine, which were not affected by theophylline treatment (Figure 4E and Figure 5E–G) [38]. Another proven side effect of theophylline treatment is the activation of histone deacetylase 2, which when in a chronic state leads to the upregulation of oxidative stress [39,40]. In the rats’ brain tissues, we noted significantly increased TBARS and oxidized glutathione levels accompanied by a reduction of reduced/oxidized glutathione ratio (Figure 5H–J) (* *p* < 0.05, * *p* < 0.05, *** *p* < 0.001). No impact of acute theophylline challenge on cholinergic neurotransmission nor astrocytic neuroinflammation markers was noted (Figure 6A–G).

### 3.4. Time-Dependent Impact of cAMP on the NAA Network

**Figure 7:**
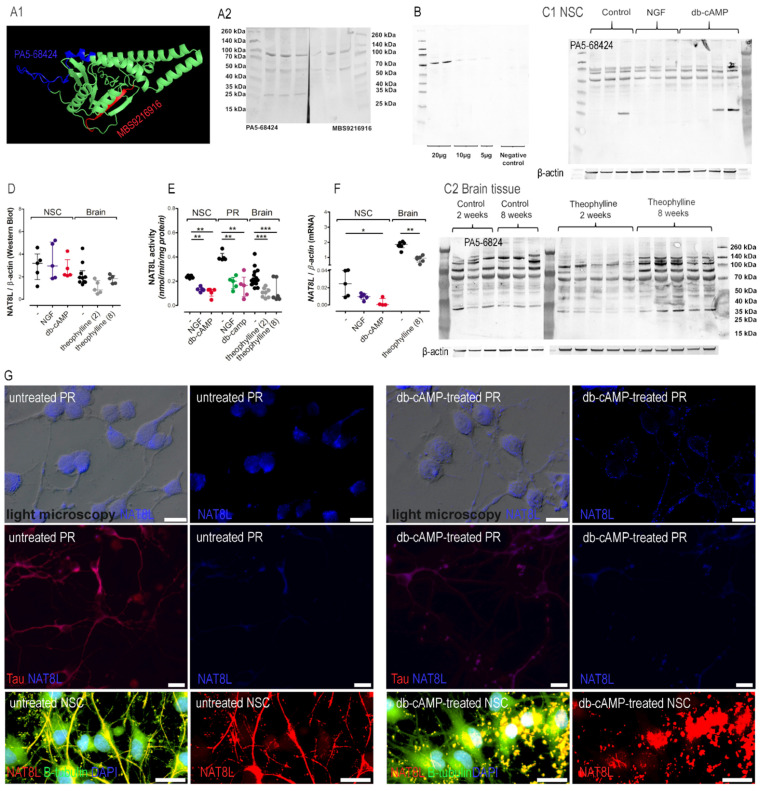
Impact of the maturation processes on the NAT8L. NAT8L tagged by PA5-68424 or MBS9216916 antibodies (**A1**) NAT8L predicted structure (PDB ID: AF-Q8N9F0-F1) with marked epitopes. Illustrations edited as per author’s permission statement, available under a CC-BY-4.0 License; (**A2**) WB membrane images of brain homogenates; (**B**) representative WB membrane images of brain homogenates or skin tissue (negative control), NAT8L tagged by PA5-68424 antibody, (**C**) representative WB membrane images of NSC (**C1**) and brain homogenates (**C2**), NAT8L tagged by PA5-68424, β-actin was used as a reference protein; (**D**) NAT8L protein level calculated from WB membrane, band: 36 kDa; (**E**) NAT8L activity; (**G**) *Nat8l* mRNA level (RT-qPCR); (**H**) fluorescence microscopy of primary neurons (PR) and neural stem cells (NSC), white scale bar represents 20 µm, images are representative for 3 independent experiments. Results are showed as medians with interquartile ranges from 4–15 observations per group. Significantly different from the control: * (*p*-value < 0.05), ** (*p*-value < 0.01), *** (*p*-value < 0.001). Abbreviations: NSC: neural stem cells; PR: primary neurons.

NAT8L protein level was analyzed in NSC and brain tissue (without cerebellum) homogenates, using the Western blot technique, with 2 commercially available antibodies targeting either the cytoplasmic domain (MBS9216916, MyBiosource, targeting residues: 59–93) or C-terminal end (PA5-68424, Thermo Fisher Sc., targeting residues 291–302) (Figure 7A1,A2). To compare these antibodies, 40 µg of protein samples (from brain tissue lysates) were loaded and developed with secondary anti-rabbit HRP-conjugated antibody (A0545, Sigma Aldrich) (Figure 7A2). The expected band size (36 kDa) was observed only with a WB membrane developed with PA5-68424 primary antibody (Figure 7A2), although both antibodies tagged bands 70 kDa and 120 kDa (Figure 7A2). First optimization study showed that 20 µg of brain tissue protein was not enough to visualize 36 kDa band characteristic for NAT8L molecular mass, thus further studies included 40 µg of protein loading (PA5-68424 antibody) (Figure 7A2–C). The same antibody did not present cross-reactivity at bands 70 kDa and 120 kDa with rat skin tissue used as a negative control (Figure 7B). Since both antibodies tagged bands 70 kDa and 120 kDa in brain lysates (but did not bind to the skin sample), we assumed that NAT8L might have been involved in a protein complex playing an unknown biological role in the mammalian brain. Our preliminary assumption was confirmed by visible changes in cellular localization (Figure 7G). Microscopic studies with fluorescence staining showed that 6 DIV treatment with db-cAMP triggered changes in NAT8L staining pattern from the cytoplasmic, regular staining covering entire neurons to grainy/dotty-like or vesicle-like staining focused close to cell nuclei (Figure 7G). Furthermore, in vivo theophylline challenge showed that chronic theophylline treatment significantly inhibits NAT8L activity, although its protein level tagged at WB membrane upper 50 kDa considerably fluctuates (Figure 7C2). Therefore, in further studies we did 2 different densitometric analyses of NAT8L protein level: we analyzed the density of 36 kDa band and analyze density results from the 70 kDa and 100 kDa (Figure 7C1,D). Such an approach has several limitations including involvements of unspecific bands. Some limitations were excluded by comparison of the WB membranes developed with primary antibodies targeting 2 different epitopes coming from 2 different and distinct NAT8L regions (Figure 7A1). Nevertheless, considering the WB limitations, we mentioned only interesting changes, which needed further assessment with high resolution techniques, e.g., proteomics. The same antibody was used to conduct WB assay as well as immunofluorescence staining (Figure 7G). In the immunofluorescence staining, the fluorescence signal comes from NAT8L-antibody complex as well as cross-reactivities, thus further techniques are needed to confirm cAMP-triggered changes in staining pattern (Figure 7G). In neural stem cells, the band size 36 kDa was noted only with a WB membrane developed from untreated cells (Figure 7C1). The absence of band size 36 kDa in cAMP or NGF- treated NSC significantly decreased the total density signal, compared to the untreated NSC (Figure 7C1,D) (* *p* < 0.05). No differences between untreated and theophylline-treated brain tissue samples were noted for band size 36 kDa (Figure 7C2,D) (*** *p* < 0.001). In 8-weeks, theophylline-treated brain tissue samples, the signals coming from bands above 70 kDa were considerably stronger (Figure 7C2). Furthermore, the cAMP-triggered differentiation of neural stem cells significantly decreased NAT8L activity and mRNA level, but did not change the *N*-acetylaspartate level (product of NAT8L activity) (Figure 4F and Figure 7E,F) (* *p* < 0.05, * *p* < 0.05, * *p* < 0.05). Theophylline challenge decreased NAT8L activity (2 and 8 weeks of daily injections) and NAT8L mRNA level (8 weeks of daily injections) (* *p* < 0.05). The *N*-acetylaspartate level was significantly decreased after 2 weeks of theophylline treatment, although prolonged lower NAT8L activity turned on the NAA restore mechanism (Figure 4F and Figure 7E) (** *p* < 0.01). Additionally, the 2-week challenge with daily injections of theophylline did not affect the levels of NAA substrates (acetyl-CoA and aspartate) or aspartate aminotransferase activity (Figure 4B–D). Furthermore, the shortage of acetyl-CoA in primary neurons did not affect NAA levels, while ATP level was significantly increased (Figure 4C,E,F) (* *p* < 0.05). These data suggested a neuronal metabolic shift to alternative energy production pathways (ATP production) and theophylline-dependent NAA level disturbances, whose pathomechanism is independent of acetyl-CoA shortage.

## 4. Discussion

Aspartate *N*-acetyltrasferase (NAT8L) has been recently discussed as an important player in psychiatric disorders [41,42,43]. By producing *N*-acetylaspartate, NAT8L regulates production of *N*-acetylaspartatylglutamate (NAAG), further used to control GABAergic neuron activity [42]. Studies with an *NAT8L*^−/−^ and *NAT8L* knock-in mouse model (microinjection with AAV into the medial prefrontal cortex), proved NAT8L involvement in location and contextual memory [41,42]. Since *N*-acetylaspartate production takes place in all neurons and requires acetyl-CoA as a substrate, in our previous projects we wondered whether NAA production affects acetylcholine neurotransmission [16,17,18]. Indeed, acetylcholine and NAA productions share a substrate, acetyl-CoA, which is an entry metabolite for the mitochondrial energy production pathway [17]. Interestingly, a 72 h treatment with dibutyryl-cAMP and retinoic acid significantly decreased *N*-acetylaspartate level and improved NAT8L protein level in SN56 cells (in vitro model of cholinergic neurons) [16]. The improvement of NAT8L level agreed with data published by Uno and colleagues in 2017 [15]. Here, a 2 h treatment with methamphetamine elevated *NAT8L* mRNA level in PC12 cells [15]. Similarly, to dibutyryl-cAMP, methamphetamine activates the cAMP-dependent protein kinase A—CREB maturation pathway [15]. Furthermore, PC12 and SN56 cells share neuronal phenotype producing cholinergic markers [15]. In contrast, a 1–5 day cAMP treatment was shown to improve *N*-acetylaspartate level in SH-SY5Y [15,44]. Eventually, different findings based on neuroblastoma studies motivated this project to elucidate the connection between *N*-acetylaspartate metabolism and maturation factors.

Neurons are known to contact with astroglia and oligoglia cells using *N*-acetylaspartate or *N*-acetylaspartylglutamate [1]. Recently, the interactions between neurons and astroglia have been given a special place in these considerations [5,15,43]. Therefore, our project starts from two different in vitro models: primary neurons (engaged to check NAA metabolism in isolated neurons) and neural stem cells. NSC present interactions between neurons and both mentioned glial cell types. The metabolic profiles significantly differ between these cell cultures. Compared to the NSC, primary neurons present higher PDHC activity accomplished with significantly lower acetyl-CoA production indicating a characteristic neuronal feature—a high rate of acetyl-CoA generation and utilization (Figure 4A,C). Furthermore, the lack of glia cells in primary neuron cultures results in neuronal NAA accumulation (Figure 4F). Interestingly, the maturation processes of primary neurons limited NAT8L activity for over 3 times, while the NAA level changes were were less dramatical (Figure 4F and Figure 7E). When neurons cultured with glia cells (NSC culture) the terns of NAA levels differs (Figure 4F). Starting from control conditions (without growth factors), NAA level is significantly lower than in primary neurons cultures. In the presence of growth factors, glia cells keep the NAA level stable (Figure 4F). NSC culture contain only nerve-origin cells (without, e.g., microglia, hematopoiesis origin cells or brain blood vessel system, which are known to transport NAA outside the brain [35]). When compare NSC culture and rats’ brain, the NAT8L activity is similar, although NAA level in brain tissue is significantly lower and fluctuates between different treatment time points (Figure 4F and Figure 7E). As a conclusion, we noted glia cells (astrocytes and oligodendrocytes) involved in the NAA level stabilization, although deeper studies should further elucidate the microglia involvement and the importance of the NAA uptake by brain blood system.

Looking further in energy metabolism, it has been shown that a mutation in *IDH1/2* genes inhibits isocitrate dehydrogenase activity and correlates with decreased NAA and NAAG levels in gliomas [45]. The authors of this paper suggest that in order to postpone the differentiation processes, malignant glioma cells block NAA and NAAG entry [45]. Other studies have shown that NAA and NAAG treatment triggers in vitro maturation of immature glia in in vitro cultures resulting in extensive astrocyte maturation [46]. In our present study, treatment with different protein kinase A activators of both NSC and rat brains significantly lowered IDH activity accomplished with decreased *NAT8L* mRNA levels and in some cases decreased NAA level (Table 4, Figure 4F and Figure 7F). Furthermore, NGF treatment did not affect these parameters. Thus, referring to the mentioned literature, we assume that protein kinase A regulates *NAT8L* gene expression in neurons, by which it has a considerably important impact on the differentiation of astroglia cells. One important issue to note is that NGF-triggered neuronal maturation strongly regulates NAA metabolism in primary neurons, although the same approach introduced to NSC results only in the regulation of NAT8L activity, without an impact on *NAT8L* mRNA or NAA levels in NSC (Figure 4F and Figure 7E,F). Thus, we assumed that the mentioned link between NAA metabolism and astrocyte maturation depends exclusively on protein kinase A activity.

The role of NAT8L and NAA metabolism is not fully elucidated, although the lack of NAA is known to increase lethality in *NAT8L*^−/−^ mice, while the chronic and extensive NAA accumulation observed in Canavan disease has a neurotoxic effect [47,48]. The overexpression of *NAT8L* in primary neuron culture indicated NAT8L as a player in ATP-dependent axonal growth [49]. Meanwhile, the knock-down of the *NAT8L* gene in primary neuron culture showed NAT8L to be responsible for neuronal elongation [50]. In *NAT8L*^−/−^ mice, the lack of NAT8L enzyme decreased dendric length as well as spine density [51]. In our present study, the immunostaining of NSC showed cAMP-dependent changes in intraneuronal NAT8L localization (Figure 7G). NAT8L was equally spread along the entire neurite, while cAMP treatment changed the saining pattern to localized near the nuclei and have a dotty-like pattern (Figure 7G). At this stage of knowledge about NAT8L, it is difficult to assume the exact role of such compartmentation, although this event corresponds with changes in WB results (Figure 7A–D). The molecular NAT8L mass is estimated as 36 kDa [52], which remained insensitive to theophylline challenge (Figure 7C2,D). However, the lysates developed with different anti-NAT8L antibodies showed extra Western blot bands occurring between 50 kDa and 100 kDa (Figure 7C2), which might correspond with the Madhavarao and colleagues’ findings, showing NAT8L involved in active biological complex with molecular mass about 670 kDa and at least 10 other bands indicating NAT8L complexes [53]. Interestingly, these bands followed the changes in NAT8L localization and changed their intensity under cAMP activator treatment (Figure 7G). Considering the mentioned literature and our findings, we assume the involvement of NAT8L in maturation following neurotransmission setup processes. Since 2- and 8-week theophylline treatment caused significant depletion in NAT8L activity and mRNA level, we checked the neurotransmission markers, but these remained unchanged (Figure 6A–D and Figure 7E,F). Furthermore, theophylline treatment did not affect the animals’ daily routines and behaviors (data not shown). In our current study, theophylline impacts markers linked with oxidative stress (decreased NADH level leading to the imbalance in NAD/NADH ratio, increased glutathione oxidation accompanying with increased TBARS and β-hydroxybutyrate levels, Figure 5 B–D,H–J), which has been noted by other researchers as well [54]. The same changes in oxidative stress markers have been noted in our previous study, in which we induced hyperglycemia in Wistar rat brains [18]. The compatible effects in diabetic rats did not affect NAA level as well as NAT8L activity or mRNA level [18]. Additionally, we induced oxidative stress in in vitro models, although the final conclusions were the same—no connection between NAA metabolism and oxidative stress [18]. Thus, we assumed that theophylline moderates NAA metabolism pathway via cAMP-dependent pathway rather than by oxidative stress.

The lack of changes in neurons might be explained by the weaker input of chronic drug treatment compared to the genetic manipulations. Another possibility is the balancing impact of glia cells in rat brains, which lack primary neuron cultures. The importance of these glial cells is obvious when comparing the NAT8L activity and NAA level in primary neurons and NSC cultures (Figure 4F and Figure 7E). In particular, a 6-day maturation of primary neurons with NGF or cAMP dramatically decreased NAT8L activity with a considerably less radical drop of NAA level (Figure 4F and Figure 7E). The same experimental conditions presented to neural stem cells showed only a moderate impact on NAT8L activity, while NAA level remained unchanged (Figure 4F and Figure 7E). To reveal astrocytes and their immunoreactivity, brain homogenates were immunoassayed with antibodies against GFAP, EAAT1 and S100β (Figure 6E–G). According to the protein levels of these markers, theophylline-triggered unstable NAA metabolism did not significantly affect astrogliosis, as it has been noted by other researchers [5,52,55,56,57].

## 5. Conclusions

In summary, our data showed that the NAT8L and entire brain NAA metabolism changes within mammalian life. It seems that NAT8L depends on protein kinase A activity and the brain microenvironment. Nevertheless, NAT8L as a protein and as an enzyme remains a not fully understood molecule with an important impact on our life.

## Figures and Tables

**Table 1:** A list of antibodies used in this project.

Target Protein	Type of Antibody	Company
β-actin	mouse primary monoclonal	Sigma Aldrich
Aspartate *N*-acetyltransferase	rabbit primary polyclonal	Thermo Fisher Sc.
Aspartate *N*-acetyltransferase	rabbit primary polyclonal	MyBioSource
β-III-tubulin	rabbit primary monoclonal	Cell Signaling
Choline acetyltransferase	rabbit primary polyclonal	MyBioSource
2′,3′-cyclic-nucleotide 3′-phosphodiesterase	mouse primary monoclonal	Sigma Aldrich
EAAT1	rabbit primary monoclonal	Santa Cruz SCBT
Glial fibrillary acidic protein	rabbit primary polyclonal	DAKO
Glyceraldehyde 3-phosphate dehydrogenase	mouse primary monoclonal	Abcam
Goat IgG	rabbit secondary polyclonal, AP—conjugated	Sigma Aldrich
Mouse IgG	goat secondary polyclonal, AP—conjugated	Sigma Aldrich
Mouse IgG1	goat secondary polyclonal, 488—conjugated	Thermo Fisher Sc.
Tau protein	rabbit primary monoclonal	ThermoFisher Sc.
Rabbit IgG	goat secondary polyclonal, AP—conjugated	Santa Cruz SCBT
Rabbit IgG	goat secondary polyclonal, 555—conjugated	Thermo Fisher Sc.
S100β	mouse	Sigma Aldrich
Synaptophysin	rabbit primary polyclonal	Abcam

**Table 2:** A list of primers and TaqMan probes used in this project.

Gene Transcript	Primers	TaqMan Probe	Transcript of Reference Gene
*Nat8l* NM_001191681.1	(F) tggctgacattgaacagtactaca (R) cacaacattgccgtccag	Universal ProbeLibrary Probe #83 (Roche, Cat #04689062001	Universal ProbeLibrary Rat *Actb* Gene Assay (Roche, Cat #05046203001)
Chat NM_001170593.1	(F) gaagcttccaagccactttc (R) gtagtagagcctcagacgacgac	Universal ProbeLibrary Probe #66 (Roche, Cat #0468851001)	Universal ProbeLibrary Rat *Actb* Gene Assay (Roche, Cat #05046203001)

**Table 3:** Metabolic profile of research models.

	Pyruvate	Lactate
Wistar rats’ brains		
Sham	14.3 (7.4–20.4)	24.5 (17.0–29.9)
Theophylline	15.5 (11.7–18.6)	21.0 (16.3–26.2)
primary neurons (PR)		
Control	33.2 (22.3–41.1)	18.8 (16.4–21.9)
NGF	11.3 (6.5–17.7)	21.3 (13.7–28.4)
db-cAMP	19.7 (11.5–28.2)	24.8 (15.9–35.2)
neural stem cells (NSC)		
Control	46.3 (29.5–78.6)	71.0 (30.7–104.2)
NGF	61.3 (31.0–89.2)	67.0 (34.5–78.3)
db-cAMP	32.2 (17.1–63.0)	51.0 (41.2–82.4)

Unit: nmol/mg protein. Results are showed as median (25th–75th percentile) from 5–12 observations per group. Significantly different from the control.

**Table 4:** Enzymatic profile of research models.

	LDH *	SC **	Aco **	IDH **
Wistar rats’ brains				
Sham	1.12 (1.07–1.34)	44.9 (33.3–55.8)	30.1 (24.5–37.1)	14.3 (12.2–17.0)
Theophylline	1.44 (1.19–1.68) *	68.0 (46.1–77.3)	28.4 (18.5–34.8)	11.2 (8.4–14.2) *
primary neurons (PR)				
Control	0.81 (0.73–0.91)	N.A.	38.3 (29.3–45.6)	81.7 (62.7–102.4)
NGF	0.88 (0.81–0.96)	N.A.	40.0 (26.6–52.0)	86.4 (65.0–106.1)
db-cAMP	0.90 (0.69–0.97)	N.A.	33.2 (21.5–45.7)	73.4 (60.7–89.2)
neural stem cells (NSC)				
Control	0.33 (0.24–0.43)	N.A.	24.0 (16.8–32.7)	56.4 (43.0–70.8)
NGF	0.29 (0.19–0.38)	N.A.	23.4 (19.0–27.3)	50.6 (38.5–61.2)
db-cAMP	0.24 (0.13–0.37)	N.A.	26.3 (18.5–33.3)	32.9 (26.5–41.1)

Unit: * µmol/min/mg protein; ** nmol/min/mg protein. Results are showed as median (25th–75th percentile) from 5–10 observations per group. Significantly different from the control: * (*p*-value < 0.05). Abbreviations: Aco—aconitase, IDH—isocitrate dehydrogenase, LDH—lactate dehydrogenase, N.A.: not applicable, SC—citrate synthase.

## Data Availability

Data is contained within the article or Supplementary Material.

## Supplementary Materials

The following are available online at https://www.mdpi.com/article/10.3390/antiox10091404/s1, Supplement 1, Table S1: A list of compounds used in this study; Supplement 2: the protocols for assays used in this study; Supplement 3: neural stem cells and primary neurons. Immunofluorescence staining.

## Abbreviations

6-CFDA: 6-carboxyfluorescein diacetate; Aco: aconitase; Asp: aspartate; AspAT: aspartate aminotransferase; b.w.: body weight; cAMP: cyclic adenosine monophosphate; ChAT: choline acetyltransferase (enzyme); Chat: choline acetyltransferase (gene); CREB: cAMP response element—binging protein; CRABP: cellular retinoic acid—binding protein; DAF-2 DA: diaminofluorescein-2 diacetate; db-cAMP: dibutyryl-cyclic AMP (cell-permeable); DIV: days in vitro; IDH: isocitrate dehydrogenase; LDH: lactate dehydrogenase; MTT: methylthiazolyldiphenyl-tetrazolium bromide; NAA: *N*-acetylaspartate; NAT8L: aspartate *N*-acetyltransferase (or Shati protein); *Nat8l*: *N*-acetyltransferase 8 Like (gene); NGF nerve growth factor; NSC: neural stem cells; PDHC: pyruvate dehydrogenase; PKA: protein kinase A; PKB; protein kinase B; PKC: protein kinase C; TBARS: thiobarbituric acid reactive substances; transRA: *trans*-retinoic acid.
