# Supplementary material for: The cAMP Inducers Modify N-Acetylaspartate Metabolism in Wistar Rat Brain
Source: Antioxidants (Basel). 2021 Sep 1;10(9):1404. doi: 10.3390/antiox10091404 (PMC8466109; doi:10.3390/antiox10091404)

Suppl. 1

Table S1. A list of compounds used in this study.

**NAME**	**COMPANY**	**Cat#**
1,4-bis(5-phenyloxazol-2-yl) benzene (POPOP)	Sigma Aldrich	P3754
1,4-Dioxan	Sigma Aldrich	292300119
2-Chloroacetamide	Sigma Aldrich	C0267
2-Thiobarbituric acid (TBA)	Sigma Aldrich	T5500
2,5-Diphenyloxazole (PPO)	Sigma Aldrich	D210404
4-Ethynylpyridine hydrochloride	Sigma Aldrich	530921
4-Nitrophenyl phosphate disodium salt hexahydrate	Sigma Aldrich	71768
6-Carboxyfluorescein diacetate (6-CFDA)	Sigma Aldrich	C5041
Acetic acid	POCH	568760114
Acetonitrile	POCH	10265456
Acetyl-CoA [acetyl-1-14-C] 50 μCi	Perkin Elmer	NEC313050UC
Adenosine	Sigma Aldrich	A4036
ADP sodium salt	Sigma Aldrich	A6646
Alcohol dehydrogenase (ADH)	Sigma Aldrich	A7011
Albumin, bovine serum (BSA)	Sigma Aldrich	A4503
AraC	Sigma Aldrich	C6645
Aspartic acid L-[U-14-C] 50 μCi	Perkin Elmer	NEC268E050UC
ATP disodium salt	Sigma Aldrich	A2383
β-hydroxybutyrole dehydrogenase	Sigma Aldrich	H9408
B27 supplement	ThermoFisher Sc	17504044
Basic fibroblast growth factor (bFGF)	ThermoFisher Sc	13256-029
BCIP	Sigma Aldrich	B6149
Boric acid	POCH	531360115
Brilliant blue G	Sigma Aldrich	27815
CHAPS	Sigma Aldrich	C9426
Choline chloride	Sigma Aldrich	C7527
cis-Aconitic acid	Sigma Aldrich	A3412
Citrate lyase (CL)	Sigma Aldrich	C0897
Citrate synthase (CS)	Sigma Aldrich	C3260

Citrate sodium dibasic	Sigma Aldrich	71635
Citric acid	Sigma Aldrich	C1909
Coenzyme A sodium salt (CoA)	Sigma Aldrich	C3144
D-Glucose	Sigma Aldrich	G5767
Diaminofluorescein-2 diacetate (DAF-2 DA)	Calbiochem	251505-M
DAPI	Sigma Aldrich	D8417
Dibutyryl-cAMP	Sigma Aldrich	D0627
Diethyl ether	Sigma Aldrich	309966
Diphenyloxazole	Sigma Aldrich	D210404
Dithiothreitol (DTT)	Sigma Aldrich	D9779
DMEM/F12 + Glutamax	ThermoFisher Sc	31331-028
DMF	Sigma Aldrich	D4551
DTNB	Sigma Aldrich	D8130
EDTA	Sigma Aldrich	E1644
Epidermal growth factor (EGF)	VWR	BDAA354010
Eserine salicylate salt	Sigma Aldrich	45720
Ethanol	Sigma Aldrich	493511
Forskolin	Sigma Aldrich	F3917
Glucose-6-phosphate dehydrogenase (G6PDH)	Sigma Aldrich	G8404
Glutamax	ThermoFisher Sc	3505006
Glutamic-Oxalacetic Transaminase (GOT)	Sigma Aldrich	G2751
Glutathione (oxidized)	Sigma Aldrich	G6654
Glutathione (reduced)	Sigma Aldrich	G6529
Glycerol	Sigma Aldrich	G2025
Glycine	Sigma Aldrich	G8898
H_3PO_4	Sigma Aldrich	79617
HBSS	ThermoFisher Sc	4442135
HCl	POCH	575283721
$HClO_4$	Fluka	77228
HEPES	Sigma Aldrich	H4034
HEPES buffer	ThermoFisher Sc	15630-056
HEPES sodium salt	Sigma Aldrich	H3784

IgG standard (from human serum)	Sigma Aldrich	I4506
Isocitrate trisodium salt	Sigma Aldrich	I1252
Isocitrate dehydrogenase (IDH)	Sigma Aldrich	I2002
α-Ketoglutarate disodium salt	Sigma Aldrich	75892
KCl	POCH	739740114
K_2HPO_4	Sigma Aldrich	P5504
KH_2PO_4	Sigma Aldrich	P5655
K_3PO_4	POCH	742020112
$KHCO_3$	Sigma Aldrich	237205
KOH	Sigma Aldrich	P5958
α-Ketoglutarate	Sigma Aldrich	75892
L-Aspartate	Sigma Aldrich	A6683
L-Glutamate	Sigma Aldrich	G5889
L-Glutamine	Sigma Aldrich	G8540
Lactic dehydrogenase (LDH)	Sigma Aldrich	L-2500
Laemmli Sample Buffer	Bio-Rad	161-0737
Laminin	ThermoFisher Sc	23017015
Lithium potassium acetyl phosphate	Sigma Aldrich	0,1409
Maleic anhydride	Fluka	63200
Malic acid	Sigma Aldrich	M1000
Malate dehydrogenase (MDH)	Sigma Aldrich	M2634
meta-Phosphoric acid (MPA)	Sigma Aldrich	M6288
Methanol	Sigma Aldrich	621995156
$MgCl_2$	Sigma Aldrich	M8266
MOPS	Sigma Aldrich	M1254
Methylthiazolyldiphenyl-tetrazolium bromide (MTT)	Sigma Aldrich	M2128
N-acetylaspartate (NAA)	Sigma Aldrich	A5625
NaCl	POCH	794121116
NAD	Sigma Aldrich	N3014
NADH	Sigma Aldrich	N8129
NADP	Sigma Aldrich	N5755
NADPH	Sigma Aldrich	N1630

NaOH	Sigma Aldrich	S8045
Nerve growth factor (NGF-β)	Sigma Aldrich	SRP4304
Neurobasal Media	ThermoFisher Sc	21103049
NH_4Cl	Sigma Aldrich	A9434
Nitrotetrazolium Blue chloride	Sigma Aldrich	N6876
Paraformaldehyde	Sigma Aldrich	158127
PBS (sterile buffer)	ThermoFisher Sc	14190086
Penicillin-Streptomycin solution	Sigma Aldrich	P4333
Phenazine methosulfate (PES)	Sigma Aldrich	P9625
Phosphotransacetylase (PTA)	Sigma Aldrich	P2783
Poly-L-ornithine	Sigma Aldrich	P4957
Protease inhibitor cocktail	Sigma Aldrich	P8340
SDS	Sigma Aldrich	L5750
Sodium oxalate	Sigma Aldrich	71800
Sodium phosphate	Sigma Aldrich	S0876
Sodium pyruvate	Sigma Aldrich	P2256
Streptozotocin	Sigma Aldrich	S0130
Sucrose	Sigma Aldrich	S9378
Ultrapure distilled water	ThermoFisher Sc	10977035
Tetrabutyloammonium bisulfate (TBAHS)	Sigma Aldrich	86853
Tetraphenylborate sodium	Sigma Aldrich	T25402
Theophylline	Sigma Aldrich	T1633
Thiamine hydrochloride	Sigma Aldrich	T4625
Toluene	Sigma Aldrich	24529
trans-retinoic acid	Sigma Aldrich	R2625
TRIS Base	Sigma Aldrich	252859
bis-TRIS	Sigma Aldrich	B9754
Triton X-100	Sigma Aldrich	T8787
Tween 20	Sigma Aldrich	P9416

Suppl. 2

The protocols for assays used in this study.

Enzymatic assays

Aconitase (Aco, EC 4.2.1.3) activity was determined using NADPH / NADP conversion technique, at λ = 340 nm and 37 °C. The reaction buffer contained 0.05 M Tris-HCl (pH = 7.4), 2 mM $MgCl_2$, 0.1 mM NADP, 1 U IDH-NADP and 100 μg of cell homogenate protein in a final volume of 0.7 mL. Enzymatic assay was initiated by the addition of 10 μL of 10 mM *cis*-aconitane (10 μL) [17-18].

Aspartate aminotransferase (GOT, EC 2.6.1.1) activity was determined using DTNB / TNB reduction technique, at λ = 412 nm and 37 °C. 1 mL of reaction buffer contained 0.1 M Tris-HCl (pH = 8.3), 1 mM sodium-EDTA, 0.018 mM acetyl-CoA, 50 mM aspartate, 0.1 mM DTNB, 2U citrate synthase (SC, EC 4.1.3.7) and 20 μg of cell homogenate protein. Enzymatic assay was initiated by the addition of 10 μL of 0.1 mM α-ketoglutarate [17-18].

Aspartate *N*-acetyltransferase (NAT8L, 2.3.1.17) activity was determined using radiochemical assay measures the level of produced [^{14}C]-*N*-acetylaspartate (^{14}C-NAA). The reaction buffer contained 10 mM potassium phosphate, 20 mM potassium-HEPES (pH = 7.1), 1 mM $MgCl_2$, 200 μM acetyl-CoA, 50 μM aspartate, 1 μM L-[U-^{14}C]aspartate and 100 μg of cell homogenate protein in a final volume of 0.2 mL (incubation conditions: 30 min, 37 °C, gentle shaking). Reaction was stopped by thermic shock (5 min, 80 °C) and dilution (1 mL of 5 mM potassium-HEPES, pH = 7.1). The final product (^{14}C-NAA) was isolated at DSC-SAX-SPE column (Discovery, Cat# 52664-U). Mobile phases: equilibration (5 mL of methanol followed by 5 mL of water and finally 5 mL of 5 mM potassium-HEPES, pH = 7.1), wash 1 (2 mL of 5 mM potassium-HEPES, pH = 7.1), wash 2 (5 mL of 0.15 M NaCl), elution (5 mL of 0.3 M NaCl). The radioactivity of eluent (2 mL) was counted for 10 min in the presence of 10 mL Ultima Gold™ liquid scintillator (Perkin Elmer, Cat# 6013321). Total radioactivity of substrate (L-[U-^{14}C]aspartate) has been measured in 1,4-dioxan-based scintillator instead [17-18].

Choline acetylaspartate (ChAT, EC 2.3.1.6) activity was determined using radiochemical assay to measure the level of produced [^{14}C]-acetylcholine (^{14}C-ACh). The reaction buffer contained 50 mM sodium phosphate buffer (pH = 7.4), 1 mM EDTA, 0.6 mM NaCl, 0.2 mM eserine, [1-^{14}C]-acetyl-CoA (0.43 nmol / sample, 0.025 μCi), 0.05 M choline and 20 μg of cell homogenate protein in a final volume of 0.01 mL (incubation conditions: 30 min, 30 °C, gentle shaking).

Reaction was stopped by dilution in ice – cold buffer having 10 mM sodium phosphate buffer (pH = 7.4), 0.5% 4-tetraphenylborate sodium, 25% acetonitrile in total volume of 6 mL. Finally, radioactive ^{14}-C ACh has been extracted in POPOP / PPO / toluene liquid scintillator and measured for 10 min. Total

radioactivity of substrate ([1-^{14}C]-acetylo-CoA) has been measured in 1,4-dioxan-based scintillator instead [17-18].

Citrate synthase (SC, EC 4.1.3.7) activity was determined using DTNB / TNB reduction technique, at λ = 412 nm and 37 ^{o}C. 1 mL of reaction buffer contained 0.1 M Tris-HCl (pH = 8.0), 0.015 mM acetyl-CoA, 0.2 mM DTNB and 20 µg of cell homogenate protein. Enzymatic assay was initiated by the addition of 10 µL of 0.2 mM oxaloacetate [17-18].

Isocitrate dehydrogenase (IDH, EC 1.1.1.42) activity was determined using NADPH / NADP conversion technique, at λ = 340 nm and 37 ^{o}C. The reaction buffer contained 0.05 M Tris-HCl (pH = 7.4), 0.6 mM $MgCl_2$, 0.5 mM NADP and 100 µg of cell homogenate protein in a final volume of 0.7 mL. Enzymatic assay was initiated by the addition of 10 µL of 10 mM isocitrate [17-18].

Lactate dehydrogenase or LDH in media assay (LDH, EC 1.1.1.27) activity was determined using NADH / NAD conversion technique, at λ = 340 nm and 37 ^{o}C. 1 mL of reaction buffer contained 0.1 M Tris-HCl (pH = 7.4), 0.2 mM NADH and 20 µg of cell homogenate protein (or 0 – 200 µL of culture media, for cell viability test assay). Enzymatic assay was initiated by the addition of 10 µL of 0.1 M pyruvate. To establish total LDH in media activity, Triton X-100 in final concentration 0.2% was added to two culture dishes with untreated cells. Dishes were incubated for the additional 2 h in usual culture conditions. Eventually, LDH in media activity from each experimental time point was divided by total LDH activity and express as a per cents of total LDH activity [17].

Pyruvate dehydrogenase (PDHC, EC 1.2.4.1.) activity was determined using cycling method [28]. In each lysate (100 µg of cell homogenate protein) the following reactions were performed: (1) citrate production, (2) citrate level measurement. The first reaction (1) was carried out in 250 µL for 30 min (37 ^{o}C, gentle shaking), buffer contained 0.1 M Tris-HCl (pH = 8.3), 2 mM $MgCl_2$, 10 mM dithiothreitol, 10 mM pyruvate, 2 mM thiamine pyrophosphate, 0.2 mM CoA, 2.5 mM oxaloacetate, 2 mM NAD, 0.15 U citrate synthase (EC 4.1.3.7). Reaction was terminated by thermic shock (10 min, 100 ^{o}C). Finally, the produced citrate level was determined using NADH / NAD conversion technique, at λ = 340 nm and 37 ^{o}C. The reaction buffer contained 0.1 M Tris-HCl (pH = 7.4), 0.1 mM NADH, 0.2 U malate dehydrogenase (EC 1.1.1.37) and 100 µL of achieved supernatant in a final volume of 0.7 mL. The assay was initiated by the addition of 10 µL of 0.1 U citrate lyase (EC 4.1.3.6) [17-18].

Metabolic assays

Acetyl-CoA level was determined using cycling method described previously [29]. In each neutralized supernatant (40 µg of cell homogenate protein) the following reactions were performed: (1) coenzyme-A removal, (2) acetyl-CoA level enhancement, (3) citrate level measurement. The first reaction (1) was carried out in 50 µL for 2 h (room temperature, gentle shaking), this reaction buffer contained 0.1 M

Tris-HCl (pH = 7.4), 1 mM maleic anhydride (dissolved in diethyl ether). The second reaction was started by the by the addition of 50 μL of second reaction buffer (50 mM Tris-HCl (pH = 7.4), 5 mM NH_4Cl, 0.01% albumin, 1.2 mM oxaloacetate, 2 mM acetyl phosphate, 1U phosphotransacetylase 0.12U citrate synthase). The 100 min lasting reaction (30 ^oC, gentle shaking) was terminated by thermic shock (10 min, 100 ^oC). Finally, the produced citrate level was determined using NADH / NAD conversion technique, at λ = 340 nm and 37 ^oC. The reaction buffer contained 0.1 M Tris-HCl pH = 7.4, 0.1 mM NADH, 0.2U MDH and achieved supernatant in a final volume of 0.7 mL. Reaction was initiated by the addition of 10 μL of 0.1 U citrate lyase (EC 4.1.3.6) [17-18].

Aspartate level was determined using NADH / NAD conversion technique, at λ = 340 nm and 37 ^oC. The reaction buffer contained 70 mM sodium / potassium-phosphate buffer (pH = 7.2), 10 mM α-ketoglutarate, 0.2 mM NADH and 100 μg of cell homogenate protein in a final volume of 0.7 mL. Reaction was initiated by the 10 μL addition of 15 U MDH and 0.6 U GOT [17-18].

ATP, ADP, AMP and adenosine levels were assayed by RP-HPLC method [31]. Briefly, supernatant (pH = 7.0) was centrifuged at Micro Spin filter (Teflon membrane, Ø 0.22 mm, CIRO, Cat# CIPT-02). 25 μL of supernatant (50 μg of cell homogenate protein) was analyzed in a pre-column protected Hypersil™ ODS C18RP column (150 x 4.6, i.d., MZ-Analysentechnik GmbH, Cat# 6045) by Flexar HPLC system (Perkin Elmer). Mobile phase A (10mM TBAHS / 100mM phosphate buffer, pH = 7.0) and mobile phase B (30% methanol) were mixed with flow rate: 1 mL/min under gradient program: 0 – 10 min (98% mobile phase A), 10 – 25 min (linear gradient form 98% to 0% mobile phase A), 25 – 40 min (0% mobile phase A), 40 – 45 (linear gradient form 0% to 98% mobile phase A). Separation time: t_{Ade} = 8.5 min, t_{ADP} = 20.0 min, t_{ATP} = 22.0 min [21].

β-hydroxybutyrate level was determined using NADH / NAD conversion technique, at λ = 340 nm and 37 ^oC. 1 mL of reaction buffer contained 0.08 M glycine-NaOH buffer (pH = 9.5), 0.2 mM NAD and 20 μg of cell homogenate protein. Reaction was initiated by the addition of 10 μL of 0.25 U β-hydroxybutyrate dehydrogenase [18].

Lactate level was determined using NADH / NAD conversion technique, at λ = 340 nm and 37 ^oC. 1 mL of reaction buffer contained 0.08 M glycine-NaOH buffer (pH = 9.5), 0.2 mM NAD and supernatant (20 μg of cell homogenate protein). Reaction was initiated by the addition of 10 μL of 0.25 U lactate dehydrogenase [18].

***N*-acetylaspartate** (NAA) level was assayed by HPLC method described previously [9]. Briefly, supernatant (pH = 2.5) was centrifuged at Micro Spin filter (Teflon membrane, Ø 0.22 mm, CIRO, Cat# CIPT-02). 90 μL of supernatant (90 μg of cell homogenate protein) was analyzed in a Synergy 4u Fusion RP 80A column (250 x 4.6, Phenomenex, Cat# 00G-4424-EO) by HPLC A-200 system (Perkin Elmer). The

assay was performed under isocratic conditions (mobile phase: 0.1% H_3PO_4 / 1% methanol / water, pH = 2.5) with flow rate: 0.9 mL/min and separation time: 20 min; t_{NAA} = 7.0 min (λ = 210 nm) [17-18].

NAD and NADH levels were determined using MTT conversion technique, at λ= 570 nm (room temperature). Supernatants obtained with 0.2 M KOH were considered as having only NADH, while deproteinized with 0.1 HCl were consider as a total amount of NAD (NAD+NADH). Hence, NAD was calculated as result of subtraction of NADH from total NAD level. The reaction buffer contained 0.1 M Tris-HCl buffer (pH = 7.4), 0.04 M EDTA. 1 mM MTT, 1 mM PES, 5 mM ethanol and supernatant (100 µg of cell homogenate protein) in a final volume of 1 mL. Reaction was initiated by the addition of 10 µL of 40 U alcohol dehydrogenases [18].

Oxaloacetate and pyruvate levels were determined using NADH / NAD conversion technique, at λ = 340 nm and 37 °C. 1 mL of reaction buffer contained 0.1 M Tris-HCl (pH = 7.4), 3 mM sodium-EDTA, 0.2 mM NADH and supernatant (100 µg of cell homogenate protein). Reaction was initiated by the addition of 10 µL of 1.5 U MDH (for oxaloacetate assay) or 4U LDH (for pyruvate assay) [17-18].

Thiobarbituric acid reactive substances (TBARS) were used to track the lipid peroxidation products at λ = 535 nm in room temperature. Briefly, 0.5 mg of cell homogenate protein was deproteinized by 10% trichloroacetic acid in a final volume of 0.6 mL (10 min, 4 °C, gentle shaking). Next, each sample was enriched by 0.2 mL of 2% thiobarbituric acid and heated for 20 min at 100 °C [17-18].

Reduced (GSH) and oxidized (GSSG) glutathione levels were determined using capillary zone electrophoresis technique described by Hempe et al. [32] with modifications. Briefly, brain tissue was homogenised in 5% *meta*-phosphoric acid (1:10, w/v) and centrifuged (15 min, 4 °C, 10 000 × g). The obtained lysates were kept at -20 °C until analysis. Each sample was diluted 10 times either with distilled water or with glutathione standards in final concentrations of 2 µM (GSH) and 0.4 µmol (GSSG). Electrophoresis was performed using P/ACE MDQ, fused silica capillary 75 µm i.d. and 57 cm long (Beckman Coulter, Cat #338473) with running buffer containing 75 mM boric acid and 25 mM bis-Tris (pH = 7.8). Samples were loaded under 0.5 psi for 20 s and run at 20 kV (normal polarity, 20 °C, 10 min) Electropherograms were analysed using Karat 32.0 (Beckman Coulter). Migration time: t_{GSH} = 5.7 min, t_{GSSG} = 6.1 min (λ = 200 nm)[22].

Proliferation and viability assays

6-CFDA test was used to calculate the total number of cell bodies being at the bottom of fluorescence – dedicated black 96-well plate. Total fluorescence emitted by 6-CFDA membrane – permeable fluorescent dye was counted using λ_{ex} = 488 nm, λ_{em} = 520 nm. The cells were seeded as usually. As soon as the cell culture was completed, 100 µL of fresh media with 2 mM Glutamax and 50 µM 6-CFDA only (without supplement or other factors) was added to each well followed by 1 h incubation in a standard

cell culture condition. Next, the wells were washed 3 times by sterile PBS. Finally, to measure the total fluorescence per each well, the cells were lysed for 15 min by lyses buffer containing 50 mM HEPES, 5 mM dithiothreitol, 0.1 mM EDTA and 0.1% CHAPS [24].

DAF-2 AM intracellular nitric oxide test was determined using diaminofluorescein-2 diacetate (DAF-2) membrane permeable fluorescent dye method with λ_{ex}=488 nm, λ_{em}=520 nm. The cells were seeded as usually, but we used fluorescence – dedicated black 96-well plates instead of regular ones. As soon as the cell culture was completed, 100 µL of fresh media with 2 mM Glutamax and 2 µM DAF-2 AM only (without supplement or other factors) was added to each well, followed by 1 h incubation at standard cell culture conditions. Next, the wells were washed 3 times with sterile PBS. Finally, to measure cellular nitric oxide linked with fluorescence dye, the cells were lysed for 15 min by lyses buffer containing 50 mM HEPES, 5 mM dithiothreitol, 0.1 mM EDTA and 0.1% CHAPS [26].

xCELLigence Real Time instrument (Roche Germany) using real time electrical impedance measurement showed the processes of well surface covering by the seeded cells. The monitoring time was 6 days with 10th min time lapses [29].

Changes in **acid phosphatase** activity were determined using *p*-nitrophenyl phosphate / *p*-nitrophenol conversion technique, at λ = 405 nm. At 96 well plate, the 0.1 mL of reaction buffer contained 0.1 M sodium-citric buffer Tris-HCl (pH = 5.4), 5 mM p-nitrophenyl phosphate, 10 µg of cell homogenate protein. Reaction was carried out for 1 h at 37 ^{o}C [25].

MTT test was used to calculate proliferation as a total mitochondria activity. Cells were seeded at 48-well plates and cultured as usually. As soon as the cell culture was completed, 0.6 mL of fresh media with 2 mM Glutamax and 5 mg/mL MTT only (without supplement or other factors) was added to each well, followed by 3 h incubation in light – protected cell culture conditions. In order to dissolve the formed formazan, the cells were lysed by 0.3 mL of lysing buffer (50% DMF, 20% SDS, pH = 4.7, overnight). Finally, the formed formazan was monitored at 690 nm [28].

A NSC: βIIItubulin GFAP DAPI

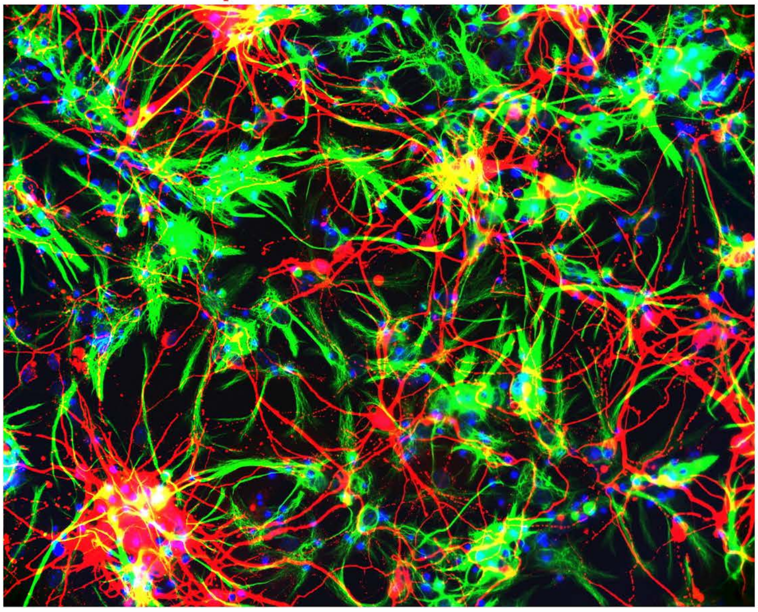

B NSC: βIIItubulin CNPase DAPI

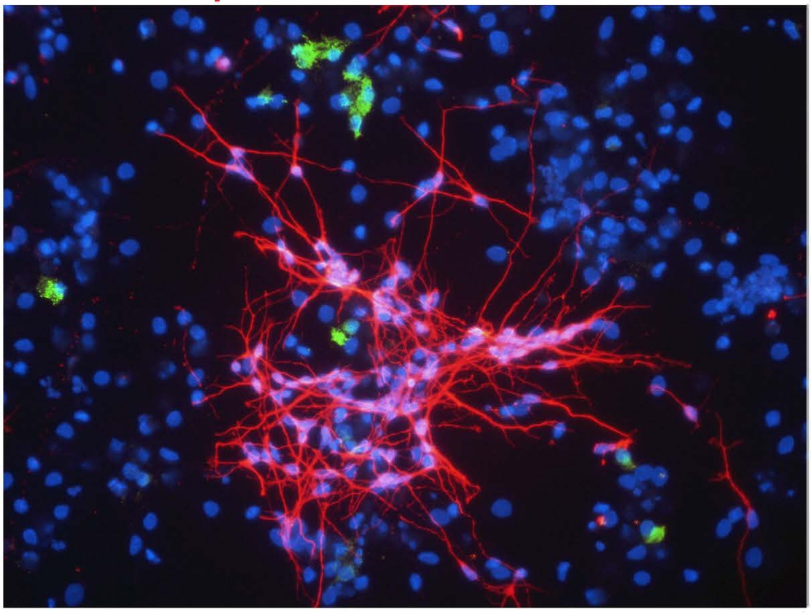

C NSC:Phalloidin GFAP

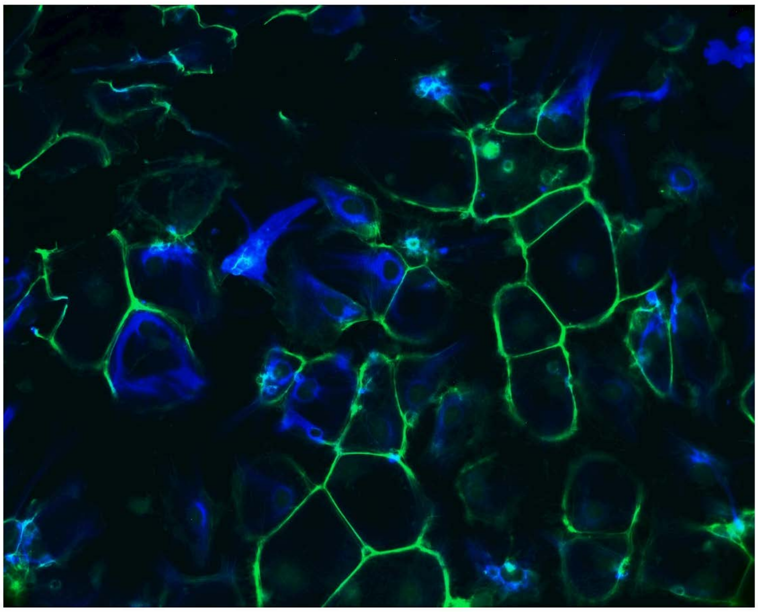

Supplement 3.

Figure S1. Neural stem cells and primary neurons. Immunofluorescene staining.

D PR: βIIItubulin

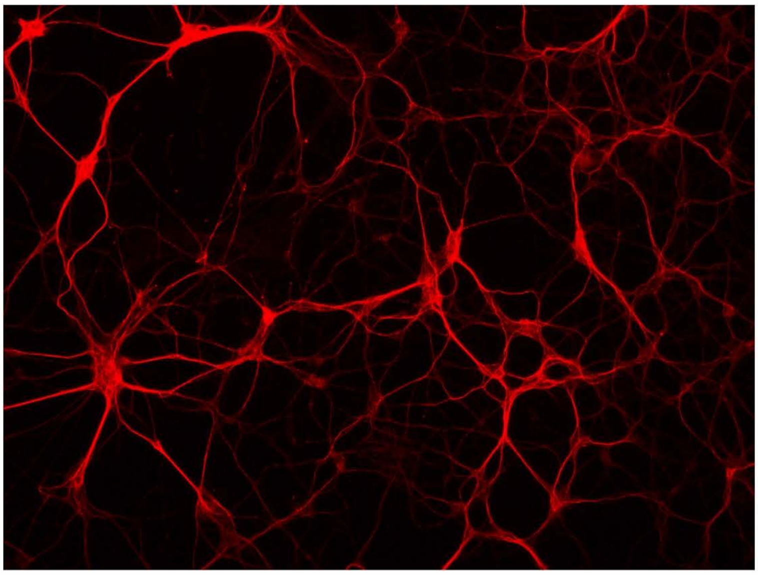

E PR: GFAP DAPI

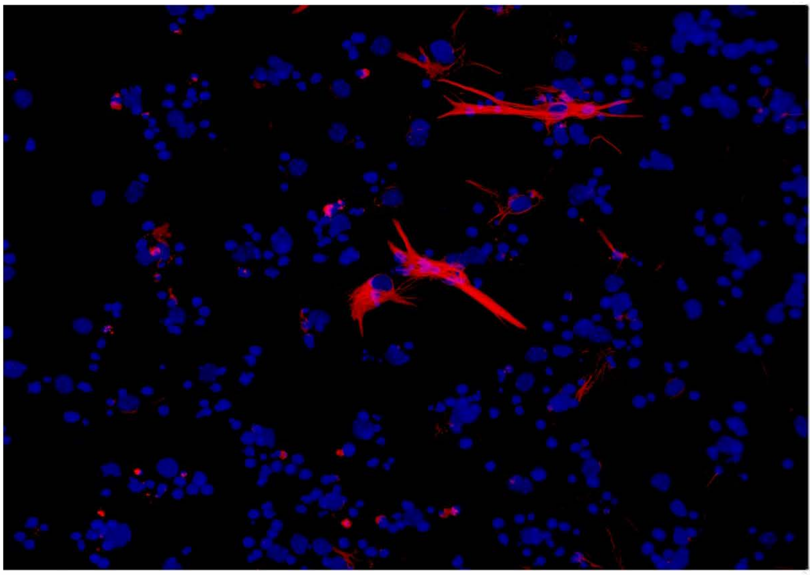

Supplement: Supplementary file 1 [file antioxidants-10-01404-s001.zip › antioxidants-1297752-supplementary.pdf]
